# Surfactant-Mediated Microalgal Flocculation: Process Efficiency and Kinetic Modelling

**DOI:** 10.3390/bioengineering11070722

**Published:** 2024-07-16

**Authors:** Carolina Maia, Vânia Pôjo, Tânia Tavares, José C. M. Pires, Francisco Xavier Malcata

**Affiliations:** 1 LEPABE—Laboratory for Process Engineering, Environment, Biotechnology and Energy, Faculty of Engineering, University of Porto, Rua Dr. Roberto Frias, 4200-465 Porto, Portugal; up201704692@fe.up.pt (C.M.); vpojo@fe.up.pt (V.P.); tsgtavares@fe.up.pt (T.T.); fmalcata@fe.up.pt (F.X.M.); 2 ALiCE—Associate Laboratory in Chemical Engineering, Faculty of Engineering, University of Porto, Rua Dr Roberto Frias, 4200-465 Porto, Portugal

**Keywords:** flocculation, gravity sedimentation, harvesting kinetics, microalgal harvesting, surfactant, *Tetraselmis* sp.

## Abstract

Microalgae are a valuable source of lipids, proteins, and pigments, but there are challenges in large-scale production, especially in harvesting. Existing methods lack proven efficacy and cost-effectiveness. However, flocculation, an energy-efficient technique, is emerging as a promising solution. Integrating surfactants enhances microalgal harvesting and disruption simultaneously, reducing processing costs. This study investigated cetyltrimethylammonium bromide (CTAB), dodecyltrimethylammonium bromide (DTAB), and sodium dodecyl sulphate (SDS) for harvesting *Tetraselmis* sp. strains (75LG and 46NLG). CTAB exhibits superior results, with 88% harvesting efficiency at 1500 and 2000 mg L^−1^ for 75LG and 46NLG, respectively, for 60 min of sedimentation—thus being able to reduce the operating time. Beyond evaluating harvesting efficiency, our study explored the kinetics of the process; the modified Gompertz model led to the best fit. Furthermore, the largest kinetic constants were observed with CTAB, thus highlighting its efficacy in optimising the microalgal harvesting process. With the incorporation of the suggested enhancements, which should be addressed in future work, CTAB could hold the potential to optimise microalgal harvesting for cost-effective and sustainable large-scale production, eventually unlocking the commercial potential of microalgae for biodiesel production.

## 1. Introduction

Microalgae are unicellular microorganisms involved in a range of applications, from wastewater treatment to the production of biofuels, besides being a source of ingredients for the food, pharmaceutical, and cosmetic industries [1,2]. These microorganisms can produce many metabolites that add value to the market, such as lipids, proteins, carbohydrates, pigments, and vitamins. Furthermore, microalgae experience rapid growth, high photosynthetic efficiency, resilience against diverse contaminants, and flexibility in terms of growing conditions [2,3]. *Scenedesmus* sp., *Chlorella* sp., and *Nannochloropsis* sp. have been the most utilised microalgal species for bioremediation and biofuel production [4]. Recently, *Tetraselmis* sp., a green marine microalga, has received much attention because of its capacity to treat wastewater and its biomass composition, which is rich in bioactive components. As this microalga has a high lipid storage capacity, its biomass can be used as a feedstock to produce biodiesel [4,5,6,7]. For instance, Teo et al. [8] reported a total lipid accumulation of 508.42 mL g^−1^ of cell dry weight in the absence of a source of nitrate. There are two types of lipids found in microalgae: nonpolar reserve lipids like triacylglycerols, and polar membrane lipids. Because of their constituents, triacylglycerols are preferred for the transesterification process in biodiesel production [8,9,10]. The lipid composition varies depending on the strain of *Tetraselmis* sp.. For example, the lipogenic strain 75LG has a higher amount of total lipids than the non-lipogenic strain 46NLG; however, the content of nonpolar lipids, ideal for producing biodiesel, is higher in the latter [11].

Before using microalgae, harvesting and disruption are necessary, to extract the desired compounds (e.g., lipids) [12]. Furthermore, the amount of biodiesel produced is determined by both the harvesting and lipid extraction procedures [9]. By using flotation with cetyltrimethylammonium bromide (CTAB) rather than centrifugation, Coward et al. [13] were able to recover more lipids suitable for producing biodiesel from microalga *Chlorella* sp. This occurred due to CTAB interactions with microalgae, which promote phospholipid bilayer solubilization and lipid extraction [13]. Moreover, the economic feasibility of microalga-based processes requires previous optimisation of their harvesting [14], and there is currently no technique for both efficient and economical harvesting [15].

Biomass harvesting represents one of the main bottlenecks in the industrial processing of microalgae, accounting for 20–30% of total biomass production [16,17]. This is quite a challenging step, because microalgae are small-sized (<30 μm), have densities similar to water, and possess a negatively charged surface that enables them to remain suspended in a medium without aggregating [15,18]. In addition, microalgae only grow well in very dilute cultures (<1 g L^−1^), to ensure sufficient light penetration. These features represent a challenge for harvesting, as the use of large volumes makes the process more complex and expensive [19]. Several conventional harvesting techniques, such as flocculation, sedimentation, flotation, membrane filtration, and centrifugation, have been suggested and thoroughly studied in the literature. An overview of the main advantages and disadvantages of all these harvesting methods is given in Table 1. Among these methods, flocculation stands out for its simplicity, effectiveness, and low energy consumption [12,16,20].

Flocculation is a complex physicochemical process in which unstable microalgae agglomerate, thus creating larger particles that settle together [23,24]. This process arises from the collision and interaction between microalgal surface charges and flocculant charges [25]. Through charge neutralisation, bridging, and sweeping mechanisms, flocculants promote the development of aggregates that settle faster in a gravitational field [12,20]. Consequently, several factors, including the type, charge, and concentration of the flocculant, the concentration and type of microalgae, the agitation, and the pH of the medium, affect the success of flocculation [23,26]. Depending on the flocculant used, this process can be classified as chemical, physical, or biological flocculation (bioflocculation and autoflocculation) [25,27].

In chemical flocculation, microalgae are harvested by adding organic or inorganic compounds bearing flocculant activity [28]. As a result, contamination occurs in the harvested biomass, which can limit the downstream processing and further use of microalgae [24]. Physical flocculation approaches, such as electroflocculation, magnetic flocculation, and ultrasonic flocculation, can avoid such contamination. Nevertheless, these techniques face a financial obstacle due to their need for energy and specific equipment [23,24,26]. In bioflocculation, microorganisms like bacteria, yeast, fungi, and algae, along with their extracellular polymeric substances (EPSs), are used to harvest microalgae. Even though chemical flocculants are avoided, microbiological contamination hinders the a posteriori use of biomass [23,24]. Moreover, this harvesting procedure is unreliable since flocculation cannot be controlled [27]. Autoflocculation entails the ability of certain microalgae to produce flocs spontaneously. This process is influenced not only by the production of EPSs by the microalgae but also by environmental conditions, such as the pH. Although it is a chemical-free and non-toxic process, autoflocculation is unsuitable for large-scale use, since it is a slow process that can only be applied to some species of microalgae [27,29]. Among these different methods, chemical flocculants have been widely used to harvest microalgae [22]. To select the appropriate flocculant (whether organic or inorganic), it is necessary to consider the microalgal species, its future applications, and the environmental impact. Therefore, organic flocculants are preferable to inorganic flocculants, since they are broken down in the environment faster, thus reducing long-term damage [25].

The ideal flocculant should be economical, effective at low dosages, sustainable, renewable, non-toxic, not susceptible to contaminating the biomass, and suitable for reuse of the culture medium [18]. Several flocculants, including synthetic and natural polymers and inorganic metal salts, have been employed to harvest microalgae via chemical flocculation [16,23]. Among them, inorganic flocculants based on iron or aluminium salts are the most popular [28]. However, the intrinsic toxicity of these inorganic flocculants poses the risk of biomass contamination, thus restricting biomass use downstream [12,20]. Furthermore, the reuse of the cultivation medium is hampered, which would otherwise prove crucial in attempts to reduce processing costs [17]. To overcome these limitations, the focus has switched to organic flocculants. Unfortunately, they are still expensive and require high dosages, so their large-scale application appears challenging [12].

The use of such surfactants as organic flocculants to harvest microalgae has been on the rise because biomass can be harvested and cells disrupted simultaneously, thus reducing costs [12,23,30]. We must remember that surfactants are molecules characterised by a hydrophilic head and a hydrophobic tail, which allow them to reduce the surface tension at liquid–liquid, liquid–gas, or liquid–solid interfaces [16,30]. When a surfactant comes into contact with microalgae, its hydrophilic head is attracted by the negatively charged cells. At the same time, its hydrophobic chain acts as an interparticle bridge between cells, thus promoting the formation of aggregates and efficient flocculation. Due to their solubilisation properties, surfactants can disrupt microalgal cells and release valuable bioproducts [12,16]. This has been confirmed by Huang and Kim [31], who showed the potential of surfactant CTAB to disrupt microalga *Chlorella vulgaris* and facilitate lipid extraction, further achieving 97% harvesting efficiency in 90 min. Other authors studied how surfactant selection influences the disruption and harvesting efficiency of the microalga *Chlorella sorokiniana*. For instance, Taghavijeloudar, Kebria, and Yaqoubnejad [12] studied two cationic surfactants, CTAB and dodecyltrimethylammonium bromide (DTAB), non-ionic triton X-100, and anionic sodium dodecyl sulphate (SDS), and the cationic surfactants were found to be more successful in promoting flocculation; regarding cell disruption, Triton X-100, and SDS were the most successful surfactants, even though all of them promoted the release of proteins and polysaccharides. These surfactants can also be used to extract bioproducts from microalga *Tetraselmis* sp., since their cell wall composition is similar to that of *Chlorella* [32,33]. Given that surfactants are toxic, using them raises environmental concerns; however, they are biodegradable [34,35,36]. Cationic surfactants can be removed using an oxygen-based membrane biofilm reactor (O_2_-MBfR) [37], while anionic surfactants can be biodegraded by bacteria [35].

Although several studies have focused on applying surfactants for harvesting and disrupting microalgae [12,16,31,34,38], their use, specifically with microalga *Tetraselmis* sp., has not yet been explored. Furthermore, no research has been conducted on the kinetics of surfactant-based flocculation processes. Our work addressed this gap, and, accordingly, aimed to optimise microalga *Tetraselmis* sp. harvesting using surfactants. For this purpose, different CTAB, DTAB, and SDS concentrations were tested for harvesting two *Tetraselmis* sp. strains (lipogenic 75LG and non-lipogenic 46NLG). The kinetics of harvesting were modelled by applying first-order, second-order, and modified Gompertz models. In summary, the main objectives of this study were to (i) evaluate the capacity of surfactants (CTAB, DTAB, and SDS) to harvest microalga *Tetraselmis* sp.; (ii) ascertain the influence of the composition of microalga (strain 75LG and 46NLG) upon the harvesting process; and (iii) assess the effect of the type and concentration of surfactants on the kinetics of the harvesting process.

## 2. Materials and Methods

### 2.1. Microalgae and Flocculants

Two different strains of microalga *Tetraselmis* sp. (75LG and 46NLG) were provided by the Microbiology Department of the Centre for Biological Research of the University of Santiago de Compostela (CIBUS). These strains were previously isolated by Prof. Ralph A. Lewin and classified as lipogenic (75LG) and non-lipogenic (46NLG) [11]. For each strain, two biological replicates, i.e., two independent cultures of each microalga strain, were used in the harvesting experiments. The microalgae were cultivated photo-autotrophically in a sterile artificial seawater medium prepared according to Darley and Volcani [39] and consisting of NaCI, 23.6 g; MgSO_4_·7H_2_O, 4.9 g; MgCl_2_·6H_2_O, 4.1 g; CaCl_2_, 1.1 g; KCI, 0.75 g; KNO_3_, 303 mg; K_2_HPO_4_·3H_2_O, 45.6 mg; Na_2_EDTA, 12 mg; thiamin·HCl, 0.5 mg; Na_2_SiO_3_·9H_2_O, 40 mg; glycylglycine, 0.66 g; and trace elements (Fe, B, 0.5 mg; Zn, 0.3 mg; Cu, Mo, Co, Mn, 0.1 mg; and Na_2_ tartrate, 1.5 mg) in 1 L of glass-distilled water. Flat-bottomed balloons with a 1 L capacity were used for growth. Said balloons were placed inside a FitoClima growth chamber (MLR-352-PE Climate Chamber, Panasonic, PHC Europe, Breda, Netherlands) and subjected to a 12 h light–12 h dark photoperiod under a light intensity of 152 μmol_photon_ m^−2^ s^−1^. Cells were inoculated at a density of 1 × 10^5^ cells mL^−1^, at an initial pH of 7.5 and temperature of 20 °C, under aeration. Cell density was monitored daily using a Neubauer haemocytometer. The optical density was measured using a spectrophotometer at 670 nm (OD_670_). As soon as cultures reached the late stationary growth phase (22 days), harvesting procedures started. These microalgal suspensions had a ca. 1 × 10^7^ cells mL^−1^ concentration.

Three different surfactants were tested in the experiments. The two cationic surfactants, cetyltrimethylammonium bromide (CTAB) and dodecyltrimethylammonium bromide (DTAB), were purchased from Sigma-Aldrich, and the anionic surfactant, sodium dodecyl sulphate (SDS), was supplied by J.T. Baker. The surfactant solutions were prepared by dissolving the necessary amount in distilled water.

### 2.2. Harvesting Experiments

The harvesting experiments started with filling up a 15 mL falcon tube with 9 mL of microalga *Tetraselmis* sp. in the late stationary phase. Then, 1 mL of the surfactant solution was added and mixed for 1 min at 300 rpm using a mini orbital shaker [17]. Surfactant concentrations of 100, 200, 300, 400, 500, 1000, 1500, and 2000 mg L^−1^ were tested. After mixing, the microalgal cells spontaneously settled for 120 min. The supernatant was collected during this period, and the OD_670_ was measured at 0, 15, 30, 60, 90, and 120 min. The harvesting efficiency (%) was calculated using Equation (1) [16]:

(1) $$Harvesting\;efficiency\;(\%)=\frac{{OD}_{670}(t_{0})-{OD}_{670}(t)}{{OD}_{670}(t_{0})}\times 100$$

*OD*_670_ (*t*_0_) and *OD*_670_ (*t*) denote the optical density at 670 nm at 0 and after *t* min, respectively. Every harvesting experiment was performed in triplicate for each biological duplicate.

### 2.3. Kinetics of Flocculation and Sedimentation

In addition to ascertaining efficiency, the kinetics of harvesting were determined. The modified Gompertz model (Equation (2)), as well as first- and second-order models (Equations (3) and (4), respectively), were employed accordingly [40].

(2) $$C\left(t\right)=C_{0}+(C_{f}-C_{0})\times exp\left[-exp\left(k\left(\lambda -t\right)+1\right)\right]$$

(3) $$C(t)=C_{0}\times exp\left(-kt\right)$$

(4) $$C(t)=\frac{1}{\frac{1}{C_{0}}+kt}$$

In the above equations, *C*_0_ and *C_f_* denote the concentration of microalgae at the beginning (*t*_0_) and end (*t_t_*) of flocculation; *k* denotes the sedimentation constant (min^−1^); and λ denotes the lag time (min).

### 2.4. Statistical Analysis

The Solver supplement in Microsoft Excel V. 2301 was used to minimise the sum of squared residuals to attempt to find the best estimates for the parameters in all kinetic models. The coefficient of determination (R^2^) was also obtained and used to assess the goodness of fit.

For each parameter, the mean value and standard deviation were determined. The statistical treatment was carried out using GraphPad Prism V. 8.0. To evaluate significant differences between means, a two-way analysis of variance (ANOVA) coupled with Tukey’s multiple comparison test was employed. The differences between results were considered statistically significant if *p* < 0.05.

## 3. Results and Discussion

### 3.1. Harvesting Efficiency

The flocculation process comprises collisions and interactions between flocculants and the microalgal cell surface [41]. Therefore, biological characteristic such as the cell wall composition are expected to play a crucial role in flocculation [42]. This work studied the harvesting efficiency of two *Tetraselmis* sp. strains (75LG and 46NLG) bearing different lipid compositions. The harvesting efficiency of the strains was assessed after 60 and 120 min of sedimentation.

#### 3.1.1. Microalga *Tetraselmis* sp. 75LG

**Figure 1:**
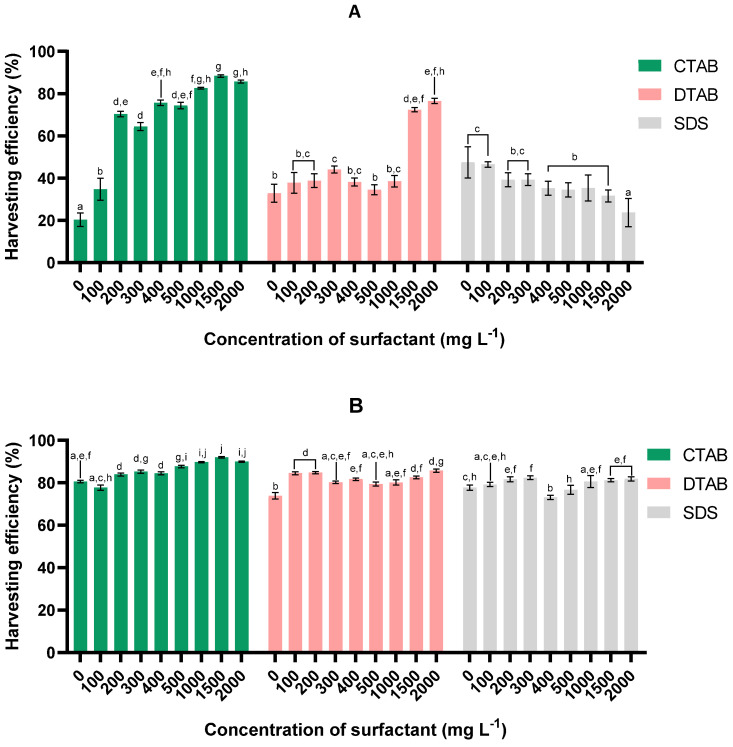
Effect of concentration and surfactant type upon harvesting efficiency in microalga *Tetraselmis* sp. 75LG after (**A**) 60 min and (**B**) 120 min of sedimentation. For each sedimentation time, values sharing at least one common letter are not statistically different (*p* > 0.05).

After 60 min of sedimentation of microalga *Tetraselmis* sp. 75LG (Figure 1A), it is apparent that the addition of CTAB enhanced the susceptibility of the microalga to harvest. A concentration of 200 mg L^−1^ of CTAB significantly increased the efficiency of the harvesting process. As the concentration of CTAB increased, the harvesting efficiency improved until reaching a maximum of 88.34 ± 0.54% under a surfactant concentration of 1500 mg L^−1^. DTAB positive effects were limited to concentrations not below 1500 mg L^−1^ (*p* < 0.05); at 2000 mg L^−1^, the highest efficiency of 76.54 ± 1.24% was achieved. SDS had no advantages; the harvesting efficiency dropped as the surfactant concentrations increased. As can be seen in Figure 1A, CTAB attained the highest harvesting efficiency. These results agree with Taghavijeloudar, Kebria, and Yaqoubnejad [12] regarding microalga *Chlorella sorokiniana* sp., with CTAB again conveying the best harvesting efficiency. Furthermore, they concluded that the application of SDS had no beneficial effects upon harvesting, while the effectiveness of DTAB was limited to high concentrations (>1000 mg L^−1^). As DTAB possesses a smaller alkyl chain than CTAB, it is less effective at promoting flocculation; i.e., it requires higher concentrations to be effective. In the case of SDS, its negative charge promotes repulsion relative to extracellular polymeric substances, which hampers flocculation and thus microalga harvesting [12].

By 120 min of sedimentation (Figure 1B), the harvesting efficiency had increased for all conditions studied and exceeded 70%, regardless of the surfactant and concentration used. For CTAB and DTAB, the highest harvesting efficiency was recorded at the same concentration for both sedimentation times (60 and 120 min); by 120 min, the highest efficiency of 92.1 ± 0.3% and 85.8 ± 0.8% was obtained for CTAB and DTAB, respectively (*p* < 0.05), with a concentration of 1500 and 2000 mg L^−1^. A positive effect of SDS was observed toward this process, with the best efficiency of 82.4 ± 0.9% obtained after 120 min of sedimentation using 300 mg L^−1^. Similar to the results for 60 min of sedimentation, the maximum efficiency was attained with CTAB, and the minimum efficiency with SDS.

By examining the harvesting efficiencies achieved after 60 and 120 min of sedimentation, we can conclude that the surfactant CTAB can significantly reduce the harvesting time. In fact, using this surfactant led to harvesting efficiencies higher than 80% after 60 min of sedimentation. In addition, after 60 min of sedimentation, harvesting efficiencies above 70% were achieved using high doses of DTAB. Even though these high efficiencies had also been reached by 120 min of sedimentation for the other conditions studied, reducing the time required to harvest microalgae appears to be crucial for process optimisation.

#### 3.1.2. Microalga *Tetraselmis* sp. 46NLG

**Figure 2:**
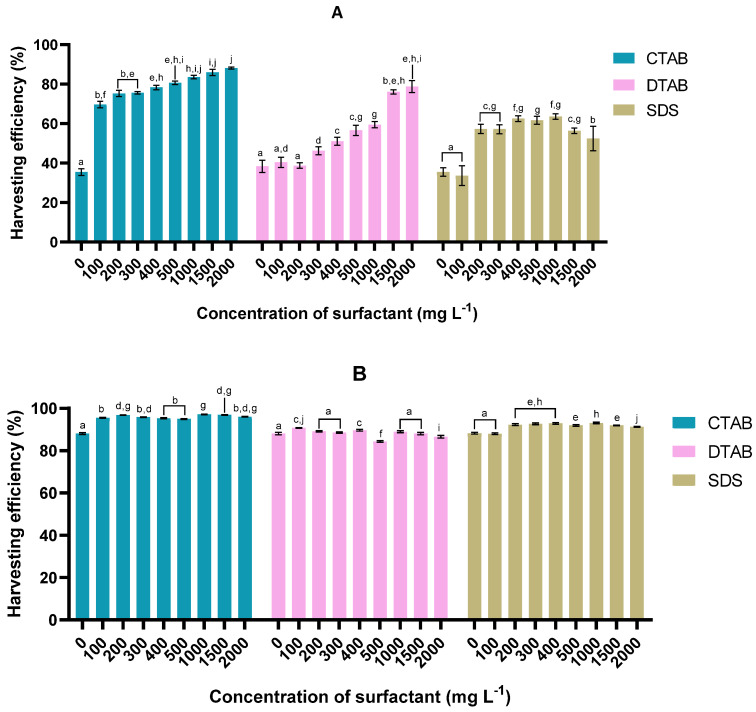
Effect of concentration and surfactant type upon harvesting efficiency in microalga *Tetraselmis* sp. 46NLG after (**A**) 60 min and (**B**) 120 min of sedimentation. For each sedimentation time, values sharing at least one common letter are not statistically different (*p* > 0.05).

In the case of strain 46NLG (Figure 2A), the best harvesting efficiency (88.2 ± 0.5%) after 60 min of sedimentation was once again obtained with CTAB, at a concentration of 2000 mg L^−1^; as the concentration of CTAB increased, the harvesting efficiency improved (*p* < 0.05).

For this strain, a DTAB concentration of 400 mg L^−1^ was already able to improve the harvesting efficiency (*p* < 0.05). This surfactant maximum efficiency of 78 ± 3% was achieved at a dosage of 2000 mg L^−1^. In the case of SDS, 400 mg L^−1^ led to the highest efficiency, i.e., 63 ± 2%; at 2000 mg L^−1^, the efficiency dropped (*p* < 0.05). This probably occurred because the flocculant was present in excess, which is known to destabilise microalgae due to electrostatic repulsion and thus delay their aggregation [43]. In fact, after 120 min of sedimentation, the harvesting efficiency for this concentration was similar to that obtained at lower SDS concentrations. Regardless of the concentration and type of surfactant, the harvesting efficiency after 120 min always exceeded 83% for this strain. Once again, the highest harvest efficiency, reaching 97%, was attained upon the addition of CTAB.

Similar to strain 75LG, the sedimentation period was shortened by adding surfactants, chiefly, CTAB. In fact, by 60 min of sedimentation, harvesting efficiencies greater than 80% had been obtained for this surfactant.

#### 3.1.3. *Tetraselmis* sp. harvesting performance

As previously reported, flocculation and harvesting are influenced by microalgal composition. Cheng, Zheng, Labavitch, and VanderGheynst [42] indeed concluded that the composition of the cell wall is an essential factor in harvesting microalgae by flocculation; better harvesting efficiencies were reported for *Chlorella* strains possessing a greater number of carbohydrates in their cell wall. As expected, the two *Tetraselmis* sp. strains used in this investigation yielded different results. By 60 min of sedimentation, the SDS surfactant had improved harvesting efficiency only for the 46NLG strain (Figure 1A and Figure 2A). Although the best efficiencies obtained by 60 min of sedimentation were identical for the other two surfactants, CTAB and DTAB (as detailed in Table S1 in Supplementary Materials), the 46NLG strain consistently yielded a better harvesting efficiency at 120 min than its 75LG counterpart. Even without surfactant, the 46NLG strain showed greater efficiency after 120 min of sedimentation and displayed greater autoflocculation ability. Once again, the rationale for such variations lies in the differences in microalgal lipid composition. High lipid concentrations increase the complexity of the harvesting process by decreasing the microalgal sedimentation capacity [27,44]. Consequently, the 46NLG strain has a higher sedimentation capacity than the 75LG strain because it contains fewer total lipids.

### 3.2. Harvesting Kinetics

When harvesting microalgae by flocculation, the efficiency of the flocculant determines how quickly flocs form and settle; in fact, the sedimentation rate increases in response to an increase in floc production [45]. The kinetics, followed by harvesting efficiency, are the key factors when searching for the ideal flocculant. This is why the kinetics of the process were also studied, with the aid of modified Gompertz, first-order, and second-order models, to further analyse harvesting efficiency.

The R^2^ values associated with the fits were examined, to ascertain which model better fitted the experimental findings. By analysing Table S2, we can see that the modified Gompertz model exhibits the best performance, with R^2^ values closest to 1, regardless of the microalga strain, type, or surfactant concentration. Therefore, only the fits for this model will be discussed hereafter.

#### 3.2.1. Microalga *Tetraselmis* sp. 75LG

**Figure 3:**
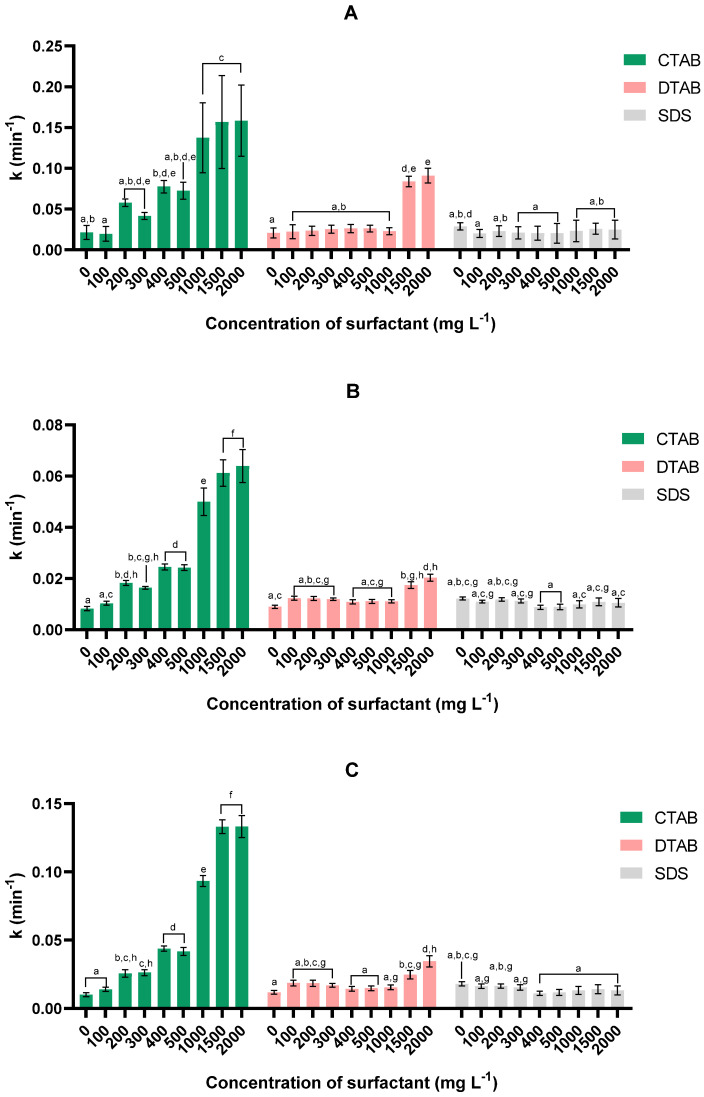
Effect of concentration and type of surfactant on sedimentation constant (*k*) as obtained via modified Gompertz (**A**), first-order (**B**), and second-order (**C**) models for microalga *Tetraselmis* sp. 75LG. For each kinetic model, values sharing at least one common letter are not statistically different (*p* > 0.05).

It was found that the trend for the sedimentation kinetic constant on the 75LG strain (Figure 3) was consistent throughout all kinetic models. It is also apparent that the highest values of this parameter were attained using surfactant CTAB. Examining the results produced by the modified Gompertz model (Figure 3A), we can conclude that the kinetic constant for CTAB increased at 1000 mg L^−1^ and essentially stabilised thereafter, with the highest value of 0.16 ± 0.04 min^−1^ being reached at 2000 mg L^−1^ (*p* < 0.05). For DTAB, this parameter only increased for concentrations equal to or greater than 1500 mg L^−1^, with the highest kinetic constant of 0.091 ± 0.04 min^−1^ being obtained at 2000 mg L^−1^ (*p* < 0.05). For the other surfactant (SDS), different concentrations did not cause statistically significant changes in this parameter (*p* > 0.05). 

When analysing in more detail the parameters obtained by the Gompertz model (Table 2), one finds that the addition of CTAB shortened the lag time, thus speeding up the harvesting process. Once again, these results corroborate the assessment drawn from the harvesting efficiency analysis. By using CTAB, achieving a high kinetic constant and a short lag time is feasible, which enables a high harvesting efficiency to be attained quickly and improves the harvesting process overall. In the case of DTAB, the lag time does not appear to have been affected by the addition of this surfactant. On the other hand, the addition of SDS caused an increase in this parameter. In fact, SDS increases repulsions because of its negative charge, which delays flocculation and, consequently, the harvesting process, as previously discussed in Section 3.1.1.

#### 3.2.2. Microalga *Tetraselmis* sp. 46NLG

**Figure 4:**
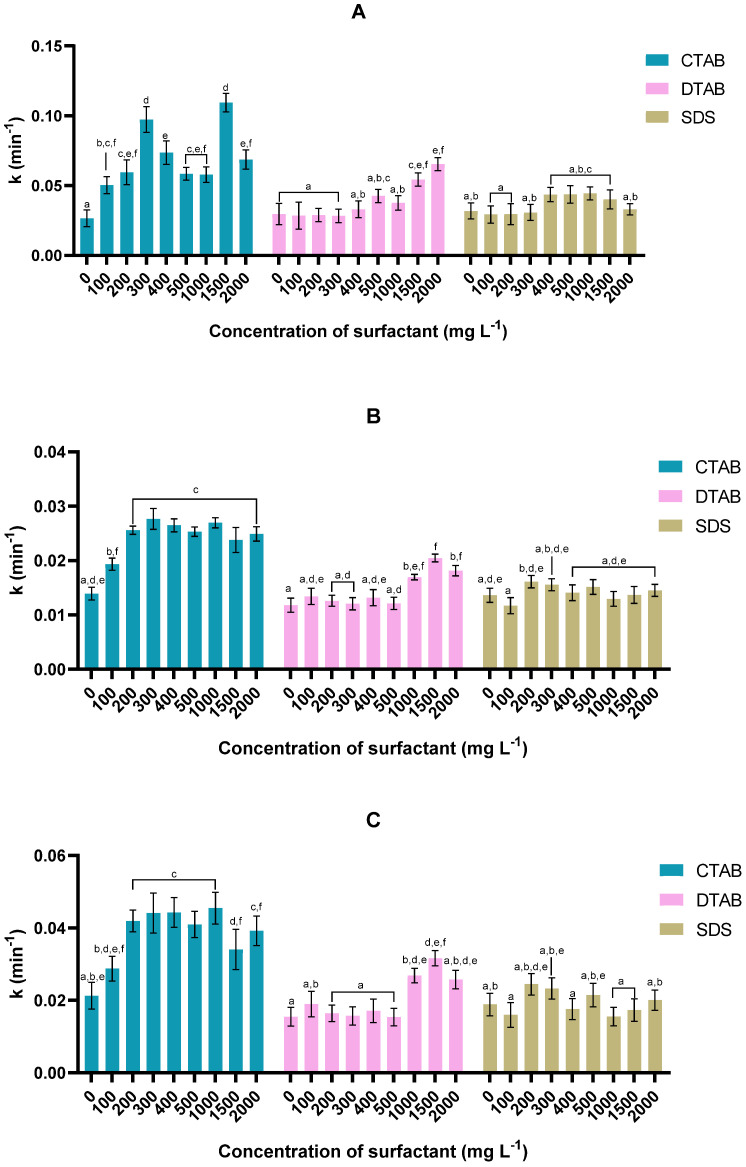
Effect of concentration and type of surfactant upon sedimentation constant (*k*) as obtained via modified Gompertz (**A**), first-order (**B**), and second-order (**C**) models for microalga *Tetraselmis* sp. 46NLG. For each kinetic model, values sharing at least one common letter are not statistically different (*p* > 0.05).

For the 46NLG strain (Figure 4), the highest *k* values were also obtained when the surfactant used was CTAB. Inspection of the modified Gompertz model (Figure 4A) indicates that the highest kinetic constants of 0.097 ± 0.009 min^−1^ and 0.109 ± 0.007 min^−1^ were obtained for CTAB concentrations of 300 and 1500 mg L^−1^, respectively (*p* < 0.05); between these concentrations, the *k* value decreased. Further inspection of Table 2 indicates that the highest lag time occurred at these two concentrations. In fact, the modified Gompertz model detected a delay at these concentrations. This led to a greater value for the kinetic constant for the conditions under scrutiny, because the same final concentration of microalgae was reached within a shorter time. This finding can be double-checked by inspection of the outcomes of the other two kinetic models (Figure 3B,C), in which the lag time was not taken into account; the *k* value increased at a concentration of 200 mg L^−1^ and then stabilised. For the other surfactants (Figure 4A), DTAB reached its maximum *k* value (0.065 ± 0.004 min^−1^) at 2000 mg L^−1^, while SDS did not show statistically significant changes in this parameter (*p* < 0.05).

Upon examining Table 2, it is clear that the addition of CTAB did not have a linear effect upon the lag time, with the highest values being obtained at concentrations of 300 and 1500 mg L^−1^. The addition of DTAB caused a decrease in lag time only for surfactant concentrations equal to or greater than 1000 mg L^−1^. In fact, analysing the harvest efficiency at 60 min of sedimentation, we see that the addition of DTAB caused more significant effects on the process at high concentrations. Regarding SDS, the addition of 200 and 300 mg L^−1^ was the only condition that allowed a reduction in lag time.

### 3.3. Future Work

The results obtained in this work emphasise the potential of CTAB as a viable flocculant for microalgal harvesting, but show that optimisation is still needed in order to reduce surfactant use and associated costs. Even though using surfactants allows for simultaneous cell rupture and harvesting, the high surfactant concentrations employed in the method may compromise its economic viability for large-scale use. Future research must thus focus on the possibility of lowering the concentration of surfactants used and/or promoting their recycling and reuse [46]. By adjusting the pH or combining the surfactants with other flocculants, such as chitosan, it may be possible to lower the concentration of surfactants needed to harvest microalga *Tetraselmis* sp. [12,16]. Furthermore, magnetic nanoparticles can be used, together with surfactants, to maximise the harvesting time and facilitate magnetic recovery while promoting the recycling and reuse of surfactants [38]. The effectiveness of these approaches for microalga *Tetraselmis* sp. (and others) will be investigated in a future study. A thorough characterisation of biomass composition will also be included in future studies, given the potential use of microalgal biomass for biofuel production and the important role of lipid content in the harvesting process.

## 4. Conclusions

Several surfactants (CTAB, DTAB, and SDS) were tested to enhance *Tetraselmis* sp. (75LG and 46NLG) microalgal harvesting. CTAB exhibited the best performance, achieving 88% efficiency within 60 min for both strains. SDS worked out only with strain 46NLG, and DTAB required high dosages (above 1500 mg L^−1^ for strain 75LG) to be effective. All conditions resulted in over 70% efficiency within 120 min. Sedimentation kinetic constants mirrored harvesting efficiencies, with CTAB producing the highest values and, accordingly, reducing the time required for efficient harvesting. Strain 46NLG showed a greater autoflocculation capacity due to its lower lipid content, an indicator of the influence of microalgal composition on flocculation. Overall, the study highlights the effectiveness of CTAB for harvesting microalga *Tetraselmis* sp., achieving high harvesting efficiencies over a short sedimentation period. Our findings suggest that CTAB could be a promising solution to the challenges of microalgal harvesting, in particular, for eventual biodiesel production.

## Figures and Tables

**Table 1:** Advantages and disadvantages of several methods used for harvesting algae [18,21,22].

Harvesting Method	Advantages	Disadvantages
Flocculation	- Fast, simple and efficient - Scalable - Reduced energy demands - Suitable for a wide variety of microalgal species - Reduced cell damage	- Difficult to separate the harvested biomass from the flocculant - Limited recycling of culture media - Biomass contamination with metals and microorganisms
Sedimentation	- Simple and inexpensive - Low energy requirements - Recycling of culture media	- Time-consuming process - Species-dependent
Flotation	- Short operation time - Low cost - Scalable - Recycling of culture media	- Need for surfactants - Species-dependent - High energy requirements
Filtration	- Chemical-free technique - Low shear stress - Recycling of water	- Membrane fouling/clogging - Frequent membrane replacement means increased expenses - Ineffective for small microalgae
Centrifugation	- Fast and efficient - Fits all microalgal types - Small-scale laboratory technique	- Expensive technique - High energy requirements - High maintenance and operation costs - Microalgal cell shearing

**Table 2:** Gompertz model parameters obtained for the microalga *Tetraselmis* sp. 75LG and 46NLG.

	Concentration (mg L^−1^)	Modified Gompertz Model			
		*Tetraselmis* sp. 75LG		*Tetraselmis* sp. 46NLG	
		*k* (min^−1^)	*λ* (min)	*k* (min^−1^)	*λ* (min)
CTAB	0	0.021 ± 0.009	20 ± 10	0.026 ± 0.006	7 ± 9
	100	0.020 ± 0.009	0 ± 22	0.05 ± 0.006	5 ± 3
	200	0.058 ± 0.005	7 ± 2	0.059 ± 0.009	0 ± 4
	300	0.042 ± 0.004	0 ± 4	0.097 ± 0.009	8 ± 1
	400	0.078 ± 0.008	0 ± 2	0.074 ± 0.008	5 ± 2
	500	0.07 ± 0.01	0 ± 4	0.058 ± 0.005	0 ± 2
	1000	0.14 ± 0.04	0 ± 6	0.058 ± 0.006	0 ± 3
	1500	0.16 ± 0.06	0 ± 7	0.109 ± 0.007	14 ± 1
	2000	0.16 ± 0.04	0 ± 5	0.069 ± 0.007	5 ± 2
DTAB	0	0.021 ± 0.006	15 ± 9	0.030 ± 0.008	20 ± 8
	100	0.022 ± 0.009	0 ± 18	0.028 ± 0.009	10 ± 11
	200	0.023 ± 0.006	5 ± 11	0.029 ± 0.005	13 ± 5
	300	0.025 ± 0.005	0 ± 9	0.028 ± 0.005	15 ± 5
	400	0.026 ± 0.005	17 ± 6	0.033 ± 0.006	18 ± 4
	500	0.026 ± 0.004	15 ± 5	0.042 ± 0.005	20 ± 2
	1000	0.023 ± 0.004	11 ± 7	0.038 ± 0.005	0 ± 6
	1500	0.084 ± 0.006	13 ± 1	0.054 ± 0.005	0 ± 3
	2000	0.091 ± 0.009	9 ± 1	0.065 ± 0.005	11 ± 1
SDS	0	0.029 ± 0.004	2 ± 7	0.032 ± 0.006	20 ± 6
	100	0.020 ± 0.005	0 ± 12	0.029 ± 0.006	20 ± 6
	200	0.023 ± 0.007	0 ± 15	0.030 ± 0.007	0 ± 11
	300	0.021 ± 0.008	0 ± 18	0.031 ± 0.006	5 ± 7
	400	0.020 ± 0.008	15 ± 13	0.044 ± 0.005	20 ± 2
	500	0.02 ± 0.01	18 ± 19	0.044 ± 0.006	20 ± 3
	1000	0.02 ± 0.01	20 ± 18	0.044 ± 0.005	20 ± 2
	1500	0.026 ± 0.007	20 ± 9	0.040 ± 0.007	20 ± 4
	2000	0.02 ± 0.01	20 ± 15	0.033 ± 0.004	14 ± 4

## Data Availability

Data available upon request due to restrictions.

## Supplementary Materials

The following supporting information can be downloaded at: https://www.mdpi.com/article/10.3390/bioengineering11070722/s1; Table S1: Harvesting efficiencies obtained for the two strains of microalga, *Tetraselmis* sp., studied; Table S2: The coefficient of determination (R^2^) obtained for the different kinetic models.
