# Supplementary material for: Surfactant-Mediated Microalgal Flocculation: Process Efficiency and Kinetic Modelling
Source: Bioengineering (Basel). 2024 Jul 16;11(7):722. doi: 10.3390/bioengineering11070722 (PMC11274027; doi:10.3390/bioengineering11070722)
Supplement: Supplementary file 1 [file bioengineering-11-00722-s001.zip › bioengineering-3069938-supplementary.pdf]

Supplementary Materials

Surfactant-Mediated Microalgal Flocculation: Process Efficiency and Kinetic Modelling

Carolina Maia [1,2,†], Vânia Pôjo [1,2,†], Tânia Tavares [1,2], José C. M. Pires [1,2,*] and Francisco Xavier Malcata [1,2]

1 LEPABE—Laboratory for Process Engineering, Environment, Biotechnology and Energy, Faculty of Engineering, University of Porto, Rua Dr. Roberto Frias, 4200-465 Porto, Portugal; up201704692@fe.up.pt (C.M.); vpojo@fe.up.pt (V.P.); tsgtavares@fe.up.pt (T.T.); fmalcata@fe.up.pt (F.X.M.)

2 ALiCE—Associate Laboratory in Chemical Engineering, Faculty of Engineering, University of Porto,
Rua Dr Roberto Frias, 4200-465 Porto, Portugal

* Correspondence: jcpires@fe.up.pt; Tel.: +351-22-041-3685

† These authors contributed equally to this work.

Table S1. Harvesting efficiencies obtained for the two strains of microalga, *Tetraselmis* sp., studied.

***Tetraselmis* sp. 75LG**				***Tetraselmis* sp. 46NLG**			
**Surfactant**	**Concentration (mg L^{-1})**	**Harvesting efficiency (%)**		**Surfactant**	**Concentration (mg L^{-1})**	**Harvesting efficiency (%)**	
		60 min	120 min			60 min	120 min
**CTAB**	0	20±3	80.6±0.6	**CTAB**	0	35±2	88.1±0.5
	1500	88.3±0.5	92.1±0.3		2000	88.2±0.5	96.1±0.1
**DTAB**	0	33±4	74±2	**DTAB**	0	38±3	88.1±0.6
	2000	77±1	85.8±0.8		2000	78±3	86.6±0.6

Table S2. The coefficient of determination (R^2) obtained for the different kinetic models.

	Concentration (mg L^{-1})	Coefficient of Determination (R^2)					
		Tetraselmis sp. 75LG			*Tetraselmis* sp. 46NLG		
		Kinetic models			Kinetic models		
		Gompertz	First order	Second order	Gompertz	First order	Second order
**CTAB**	0	0.925	0.830	0.760	0.987	0.953	0.882
	100	0.945	0.923	0.867	0.984	0.965	0.899
	200	0.992	0.968	0.911	0.979	0.989	0.960
	300	0.990	0.985	0.946	0.986	0.965	0.925
	400	0.991	0.968	0.982	0.982	0.974	0.933
	500	0.987	0.976	0.967	0.994	0.988	0.945
	1000	0.995	0.946	0.995	0.994	0.990	0.953
	1500	0.995	0.970	0.997	0.993	0.921	0.851
	2000	0.998	0.962	0.994	0.988	0.974	0.935
**DTAB**	0	0.970	0.926	0.870	0.951	0.863	0.778
	100	0.955	0.951	0.905	0.956	0.912	0.838
	200	0.982	0.955	0.895	0.978	0.919	0.835
	300	0.978	0.975	0.928	0.984	0.923	0.843
	400	0.972	0.914	0.842	0.981	0.905	0.811
	500	0.984	0.930	0.851	0.984	0.898	0.814
	1000	0.981	0.944	0.879	0.980	0.987	0.944
	1500	0.992	0.942	0.884	0.990	0.982	0.950
	2000	0.992	0.969	0.939	0.991	0.958	0.894
**SDS**	0	0.983	0.968	0.909	0.976	0.900	0.809
	100	0.984	0.965	0.911	0.984	0.893	0.816
	200	0.958	0.947	0.891	0.957	0.937	0.873
	300	0.979	0.962	0.905	0.967	0.936	0.862
	400	0.964	0.915	0.854	0.976	0.849	0.743
	500	0.963	0.912	0.848	0.968	0.895	0.803
	1000	0.953	0.885	0.811	0.979	0.842	0.738
	1500	0.987	0.896	0.808	0.956	0.830	0.726
	2000	0.964	0.870	0.782	0.984	0.931	0.855
